# The Effects of Krill Oil on mTOR Signaling and Resistance Exercise: A Pilot Study

**DOI:** 10.1155/2018/7625981

**Published:** 2018-04-26

**Authors:** John Georges, Matthew H. Sharp, Ryan P. Lowery, Jacob M. Wilson, Martin Purpura, Troy A. Hornberger, Flint Harding, James H. Johnson, David M. Peele, Ralf Jäger

**Affiliations:** ^1^Applied Science and Performance Institute, 5850 W. Cypress St., Tampa, FL 33607, USA; ^2^Increnovo LLC, 2138 E. Lafayette Pl, Milwaukee, WI 53202, USA; ^3^Department of Comparative Biosciences, University of Wisconsin-Madison, Madison, WI 53706, USA; ^4^Avoca Inc., 841 Avoca Farm Rd., Merry Hill, NC 27957, USA

## Abstract

**Introduction:**

Krill oil supplementation has been shown to improve postexercise immune function; however, its effect on muscle hypertrophy is currently unknown. Therefore, the aim of present study was to investigate the ability of krill oil to stimulate mTOR signaling and its ability to augment resistance training-induced changes in body composition and performance.

**Methods:**

C_2_C_12_ myoblasts cells were stimulated with krill oil or soy-derived phosphatidylcholine (S-PC), and then, the ratio of P-p70-389 to total p70 was used as readout for mTOR signaling. In double-blind, placebo-controlled study, resistance trained subjects consumed either 3 g krill oil daily or placebo, and each took part in an 8-week periodized resistance training program. Body composition, maximal strength, peak power, and rate of perceived recovery were assessed collectively at the end of weeks 0 and 8. In addition, safety parameters (comprehensive metabolic panel (CMP), complete blood count (CBC), and urine analysis (UA)) and cognitive performance were measured pre- and posttesting.

**Results:**

Krill oil significantly stimulated mTOR signaling in comparison to S-PC and control. No differences for markers on the CMP, CBC, or UA were observed. Krill oil significantly increased lean body mass from baseline (*p*=0.021, 1.4 kg, +2.1%); however, there were no statistically significant differences between groups for any measures taken.

**Conclusion:**

Krill oil activates mTOR signaling. Krill oil supplementation in athletes is safe, and its effect on resistance exercise deserves further research.

## 1. Introduction

Athletes use nutrition strategies to improve their training and performance through increasing their metabolic capacity, delaying the onset of fatigue, and improving muscle hypertrophy by enhancing recovery, improving immune function, and decreasing oxidative stress. Krill oil is rich in long-chain omega-3 polyunsaturated fatty acids (PUFAs), eicosapentaenoic acid (EPA), and docosahexaenoic acid (DHA), which have been found to have positive effects on inflammation [1]. In krill oil, omega-3 PUFAs are bound to phospholipids (PL), whereas in fish oil, the majority of omega-3 PUFAs are bound to triacylglycerol (TG) [2]. Greater bioavailability of omega-3 PUFA from krill oil in comparison to fish oil has been suggested based on lower doses of krill oil needed to result in a similar bloodstream level of EPA and/or DHA; however, more carefully controlled human trials are needed to establish their relative efficacies after chronic administration [3]. Contrasting results have been found on the effects of fish oil supplementation on muscle damage and postexercise soreness in healthy men and women. While most of studies at ranges from 2.4 to 6 g/d for 1 to 8 weeks have shown beneficial effects [4–8], two studies showed no effect [9, 10], potentially due to different types of exercise used to induce muscle damage or differences in the dose and duration of omega-3 PUFA supplementation among studies. A combination of EPA and DHA was found to increase the rates of muscle protein synthesis via an increase in activation of the mTOR-p70s6k signaling pathway in young- and middle-aged men and women [11]. Fish oil supplementation in combination with [12] or without [13] resistance exercise resulted in increased strength and functional ability in older adults. However, potential long-term benefits of omega-3 PUFA supplementation on muscle hypertrophy and performance in young healthy subjects undergoing a controlled resistance training program are currently unknown.

Krill oil contains astaxanthin, a red carotenoid pigment and strong antioxidant that naturally occurs in salmon, shrimp, krill, crustaceans, or certain types of algae, giving krill its reddish color. Astaxanthin administration has been shown to reduce muscle damage [14, 15], to increase time trial performance and power output in competitive cyclists [16], and to increase strength/endurance (number of squats) [17]. However, astaxanthin failed to improve muscle soreness and muscle damage in resistance trained men following an acute bout of eccentric exercise (coadministered with lutein) [18]. Discrepancies in astaxanthin's ability to attenuate exercise-induced muscle injury might be due to the type of exercise stress (resistance or endurance exercise) or the dosage and timing of the administration. Krill oil contains approximately 0.5 mg of astaxanthin per 3 g of krill oil, which is below the currently established effective dose of 4 mg for athletes. However, the absorption of astaxanthin can be greatly enhanced in the presence of fats, surfactants, or phospholipids. The phospholipid content in krill oil will likely increase the absorption of astaxanthin closing the gap between the actual content and effective dose.

In athletes, krill oil has been shown to improve postexercise immune function (2 g/d for six weeks) [19] and diminished postexercise oxidative damage during recovery (1 g/d for six weeks) [20]; however, it failed to improve exercise performance (cycling time trial [19] and total run time in a 2,000 m test [20]). The lack of performance benefits of krill oil supplementation in previous exercise studies might have been based on a lack of an accompanying controlled challenging training protocol optimizing krill oil's benefits on recovery, as muscle recovery after an exercise bout might influence training adaptations. While krill oil's effect on mTOR signaling is currently unknown, DHA/EPA has been shown to activate the mTOR-p70s6k signaling pathway [11]. A comparison of soy-derived (containing no omega-3 PUFAs) and PUFA-containing egg-derived phosphatidic acid showed a potential attenuating effect of PUFAs bound to PL on mTOR activation [21]. Therefore, the aim of the present study was to investigate the ability of krill oil to stimulate mTOR signaling and its ability to augment resistance training-induced changes in body composition and performance.

## 2. Materials and Methods

### 2.1. Cell Culture Tests

C_2_C_12_ myoblasts (ATCC; Manassas, Virginia) were plated at approximately 30% confluence and grown for 24 hours in 10% FBS high glucose DMEM with antibiotics (100 *μ*g/ml streptomycin and 100 U/ml penicillin; Sigma). At 16 hours prior to the experiment, myoblasts cells were switched to serum-free high-glucose DMEM (no antibiotics) and were approximately 70% confluent at the time of the experiment with no indication of myoblast fusion present. All stimulants were dissolved in chloroform to yield a concentration of 10 mg/mL. Each stimulant was then dried with a stream of nitrogen gas and resuspended in PBS to obtain either 20 or 60 nmol/100 *µ*L, such that 100 *µ*L added to 2 mL of media resulted in 10 or 30 *µ*M, respectively. Accordingly, cells were stimulated for 20 minutes with vehicle (Control; 100 *µ*L of PBS), 10 or 30 *µ*M of krill oil (Rimfrost USA, Merry Hill, North Carolina, USA) or S-PC (Avanti Polar Lipids, Inc., Alabaster, Alabama, USA) as the negative control. Cells were then harvested in lysis buffer (40 mM Tris, pH 7.5; 1 mM EDTA; 5 mM EGTA; 0.5% Triton X-100; 25 mM *β*-glycerophosphate; 25 mM NaF; 1 mM Na_3_VO_4_; 10 *µ*g/mL leupeptin; and 1 mM PMSF) and subjected to immunoblotting. Equivalent amounts of protein from each sample were dissolved in Laemmli buffer and subjected to electrophoretic separation on 7.5% SDS-PAGE. Following electrophoretic separation, proteins were transferred to a polyvinylidene difluoride membrane in transfer buffer (242 mM Tris, 58 mM glycine). Membranes were blocked with 5% powdered milk in TBST (Tris-buffered saline with 0.1% Tween 20) for 1 h followed by an overnight incubation at 4°C with rabbit anti-phospho-p70 S6 kinase (P-p70(389); Cell signaling #9234; 1 : 1000) dissolved in TBST containing 1% BSA. Membranes were washed for 30 min in TBST and then incubated for 1 h at room temperature in 5% milk-TBST containing peroxidase-conjugated anti-rabbit (PI-1000 Vector Laboratories, Burlingame, CA, USA). After 30 min of washing in TBST, the blots were developed on a Chemi410 camera mounted to a UVP AutoChemi system (UVP, Upland, CA, USA) using prime-enhanced chemiluminescence (ECL) reagent (Pierce; Thermo Fisher Scientific, Rockford, IL, USA). Once the appropriate image was captured, the membranes were stripped for 30 minutes in stripping buffer (100 mM *β*-mercaptoethanol, 2% SDS, 62.5 mM Tris-HCl pH 6.8) maintained at 50°C. Membranes were washed with TBST and then reblocked with 5% powdered milk in TBST for 1 h followed by an overnight incubation at 4°C with rabbit anti-p70 S6 kinase (total p70, cell signaling #2708, 1 : 2000) dissolved in TBST containing 1% BSA. Membranes were washed for 30 min in TBST and then incubated for 1 h at room temperature in 5% milk-TBST containing peroxidase-conjugated anti-rabbit. After 30 min of washing in TBST, the blots were developed on UVP using a regular ECL reagent. Once the appropriate image was captured, the membranes were stained with Coomassie blue to verify equal loading in all lanes. Densitometric measurements were performed by determining the density of each band using the public domain ImageJ software (U.S. National Institutes of Health, Bethesda, MD, USA; http://rsb.info.nih.gov/nih-image/). The ratio of P-p70(389) to total p70 was used as a readout for mTOR signaling.

### 2.2. Human Efficacy Study

#### 2.2.1. Study Design

This study consisted of a randomized, double-blind protocol consisting of 2 groups of individuals given either 3 g of placebo (olive oil) or 3 g of krill (*Euphausia superba*) oil (Rimfrost Sublime, Rimfrost USA, LLC, Merry Hill, NC, USA, Lot 8723-15-01-03, consisting of 43.8% phospholipids (1.3 g), delivering 963 mg total omega-3 fatty acids (240 mg DHA, 393 mg EPA), and 0.54 mg astaxanthin) pre-workout on training days, and with breakfast on nontraining days, during an 8-week high intensity, nonlinear, periodized resistance training protocol in form of 6 500 mg softgel capsules. The softgel capsules were manufactured by Aenova, Miami, FL, USA. Body composition, maximal strength, peak power, and rate of perceived recovery were assessed collectively at the end of weeks 0 and 8. In addition, safety parameters (comprehensive metabolic panel (CMP), complete blood count (CBC), and urine analysis (UA)) were measured pre- and posttesting.

#### 2.2.2. Subjects

**Figure 1:**
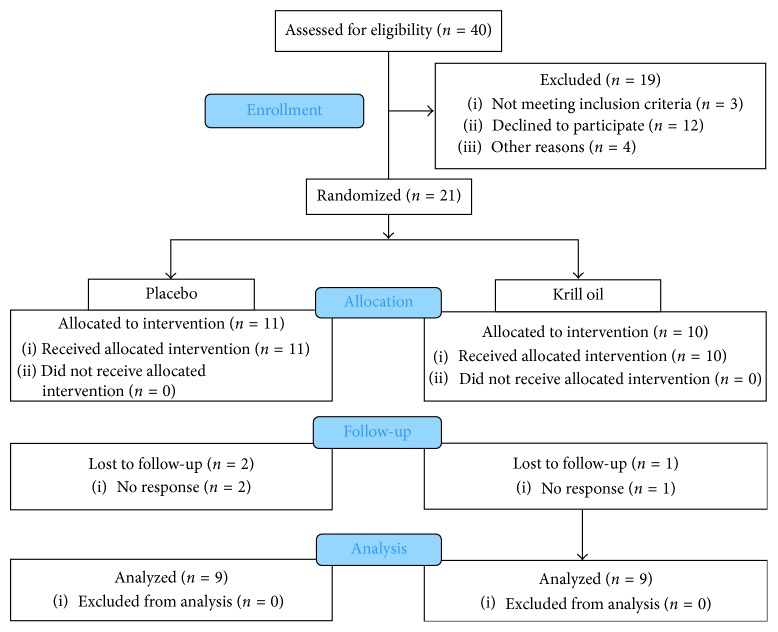
CONSORT chart.

Forty subjects were assessed for eligibility and of the twenty-one subjects enrolled 11 subjects received the placebo and 10 received krill oil. A total of 3 subjects were lost to follow-up by a lack of communication with the researcher (2 from the placebo and 1 from the krill oil group). A total of 18 subjects were analyzed, 9 from each condition (Figure 1).

Subject inclusion criteria were males 18 to 30 years of age, resistance training at least 2 times per week for the past six months, a minimum of 1 year of training experience active and currently resistance training, free of musculoskeletal, metabolic, and respiratory disorders, free of cardiovascular disease, no musculoskeletal injuries with the last six months, no history of smoking or drug use, no history of excessive alcohol consumption, not taking prescription medication, have not used a fish oil-, thermogenic-, protein-, amino acid-, or creatine supplement within the prior two months, and have not habitually used caffeine (e.g., more than 2 cups of coffee per day). Subjects were matched-paired by age, body mass, strength and resistance training, and physical activity background and then randomly placed into one of the two groups. Subjects were prohibited from consuming any nutritional supplements (including fish oil) for the duration of the study. Additionally, subjects were instructed to avoid consumption of all fish and fish byproducts. After an explanation of the procedures and associated risks, all volunteers completed written informed consent. All procedures were approved by the IntegReview Institutional Review Board, Austin, TX, USA (protocol #7952). This study was registered with the ISRCTN registry (ISRCTN11524409). Subject characteristic data are displayed in Table 1.

#### 2.2.3. Resistance Exercise Training Protocol

Resistance training occurred four days per week (programmed, nonlinear training split). The resistance training protocol was modified from Kraemer et al. [22] and Montiero et al. [23]. These researchers found that a nonlinear resistance training program yielded greater results than a traditional or nonperiodized program in athletes (Montiero et al. [23]). The program was designed to train all major muscle groups using many compound movements for the upper body (e.g., bench press, dips, shoulder press, pull-ups, and bent over rows), lower body (leg press, leg extensions, and glute-ham raises), and the core. The programmed, nonlinear training split was divided into hypertrophy days consisting of 8–10 RM loads for 3 sets, with 90 seconds rest, strength endurance days consisting of 12–15 repetitions, with 60 seconds rest, and on days training maximal strength consisting of 3 to 5 RM loads with 3 sets for all exercises except the leg press and bench press (5 total sets). Weights were progressively increased or decreased on a set-by-set basis by 2–5% to meet prescribed repetitions. Briefly, when subjects could have achieved more than 2 repetitions over the prescribed number, the load was increased by 2–5%. When subjects failed to reach the prescribed repetitions under their own will, the load was decreased by 2–5%. All training sessions were closely monitored to ensure effort and intensity to maximal on each training sessions. Subjects trained each body part twice weekly and alternately between hypertrophy and heavy workouts. We have selected this protocol because a meta-analysis by Rhea [24] indicated that training at this frequency was ideal for moderately resistance trained individuals (Table 2).

#### 2.2.4. Body Composition

A whole-body dual-energy X-ray absorptiometry (DXA) (Hologic, Bedford, MA, USA) scan was utilized to measure body composition. Lean body mass (LBM) and fat mass (FM) were determined for the total body with the subject laying in a supine position with the knee extended and instructed not to move for the entire duration of the scan (∼5 minutes). Results from each scan were uploaded and accessed on a computer directly connected to the DXA device. All DXA scans were conducted prior to and after the completion of the study, and each subject was required to fast overnight (10 hours) prior to the DXA scan. Calibration of the DXA device was done against a phantom provided by the manufacturing company prior to testing.

#### 2.2.5. Strength Assessment

One-repetition maximum (1 RM) was assessed on bench press and leg press at baseline and after 8 weeks. Loads were increased incrementally until maximal load or failure at a given load was reached. Briefly, subjects performed a general warmup and a specific warmup consisting of three sets. During the first set, subjects performed 10 repetitions with 50% of their predicted 1 RM. For the second set, they performed five repetitions with 70% of the predicted 1 RM. In the third set, subjects perform one repetition with 90% of their predicted 1 RM. After the completion of warmup sets, subjects rested for 3 minutes. Then, each subject had as many as five attempts to achieve their 1 RM load with 3–5 minutes rest between each attempt.

#### 2.2.6. Perceptual Measures

The perceptual measures were collected using a perceived recovery status scale. Ratings of perceived recovery were collected at the beginning and end of every week. The perceived recovery status scale consisted of a scalar representation numbering from 0 to 10. Visual descriptors of “very poorly recovered,” “adequately recovered,” and “very well recovered” for perceived recovery were presented at numbers 0, 5, and 10, respectively. Subjects were asked to identify their level of perceived recovery after warming up and before performing the training protocol.

#### 2.2.7. Stroop Test

The Stroop test is a psychological test of mental vitality and flexibility. The test consists of computerized presentation of the names of four colors (yellow, blue, green, and red), written in capital letters, Times New Roman font, size 24, in black. The order is semirandom, so that the same word never appears two consecutive times throughout the test. The subject's task is to read each word as quickly as possible. This part of the test is intended to obtain a baseline to evaluate the reading ability and determine whether this ability is high enough so as not to hinder the interference effect. This is because the effect of color-word interference may be absent if the reading ability is lower than expected. Characterized by a series of colorized words, which themselves are colors, subjects were instructed to read the color of the word aloud and not the word itself. Correct answers and time to completion were recorded. “Congruent” indicates the number of correct responses out of five responses to the test with matching words and colors. “Incongruent” indicates the number of correct responses out of fifteen responses to the test with nonmatched words and colors.

#### 2.2.8. Blood Safety Markers

Subjects donated approximately 10 mL of fasted, whole blood at the baseline and at the end of week 8. All blood samples were collected by a trained phlebotomist via venipuncture of an antecubital vein in the forearm using standard sterile procedures. Biomarkers comprising CMP, CBC, and lipid panels were assayed for data collection.

#### 2.2.9. Statistical Analysis

Before carrying out the parametric statistical analysis, dependent variables were examined for a normal distribution and outliers through investigation of boxplots and a normality test (i.e., Shapiro–Wilk). Normality tests revealed no outliers, and analysis was performed using the original data set. Repeated measures ANOVA were used to scrutinize the effects of supplementation on dependent variables assuming the group (placebo and krill oil) and time (pre- and posttest) as fixed factors (GraphPad Prism 7®, La Jolla, CA). Whenever an interaction or main effect was demonstrated, a Bonferroni post hoc test was performed to identify where the differences occurred. The significance level was previously set at *p* < 0.05. All data are reported as mean ± SEM. Analysis of baseline characteristic revealed that there were no statistical significant difference between groups for the investigated dependent variables (*p* > 0.05).

## 3. Results

### 3.1. Cell Culture Tests

**Figure 2:**
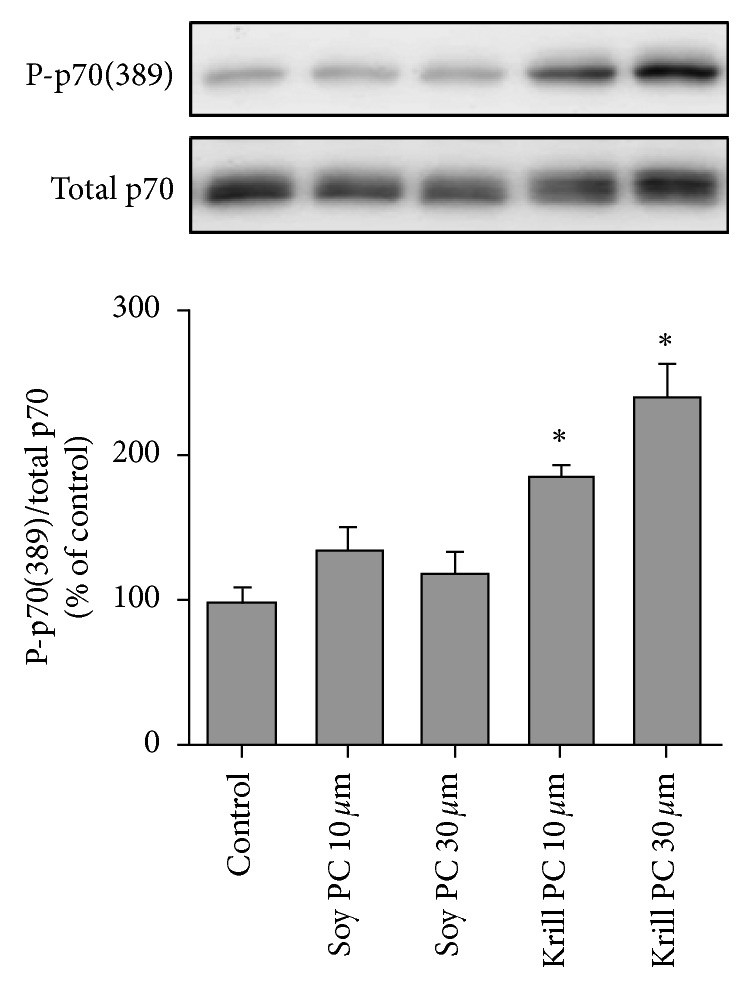
The effect of phosphatidylcholine on the activation of mTOR signaling. C_2_C_12_ myoblasts were stimulated for 20 minutes with vehicle (control), or 10–30 *µ*M of S-PC, or krill oil-derived PC. The samples were then subjected to the western blot analysis for p70 phosphorylated on the threonine 389 residue (P-p70(389)) and total p70. The ratio of these signals was calculated and used as a marker of mTOR signaling. Values in the graphs represent the mean + SEM and were obtained from 2 to 3 independent experiments (*n* = 3–6/group). ^∗^Significantly different from the control and S-PC (*p* < 0.05).

As shown in Figure 2, S-PC showed no increase in the ratio of P-p70-389 to total p70 compared to vehicle (control) stimulated cells. In contrast, elevated mTOR signaling was observed at all tested concentrations of krill oil-PC (+187% and +242% resp.; *p* < 0.001).

### 3.2. Human Efficacy Study

#### 3.2.1. Body Composition

**Figure 3:**
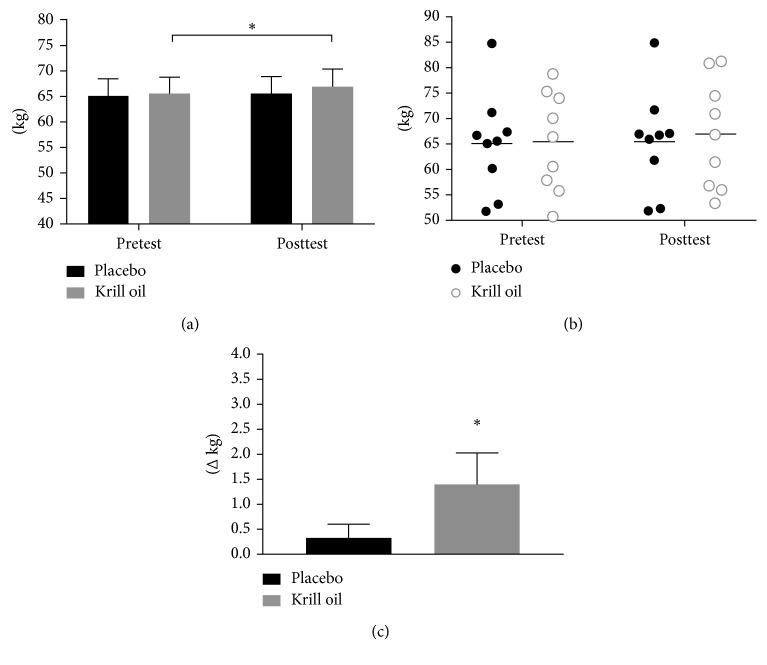
Summary of the group lean mass values (a), individual lean mass response (b), and lean mass delta change (c) (^∗^significant within-group difference (*p* < 0.05)).

A significant main time effect was noted for lean mass (*p*=0.021); however, there were no differences between the groups (placebo: mean_diff_ 0.3 kg, +0.5%; krill oil: mean_diff_ 1.4 kg, +2.1%, Figure 3). Regarding the main time effect for lean mass, post hoc analysis indicated a within-group difference from pre-to posttesting for krill oil (*p*=0.024, Figure 3(a)).

**Figure 4:**
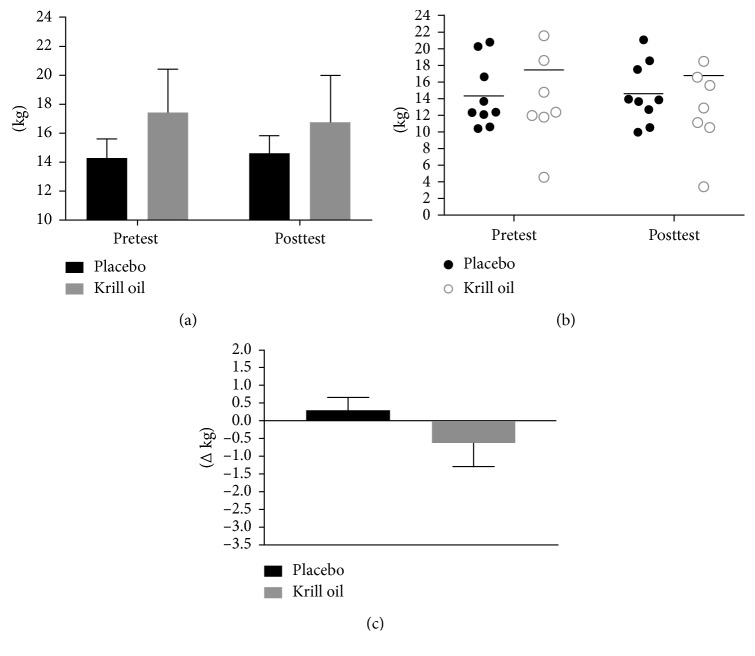
Summary of group fat mass values (a), individual fat mass response (b), and fat mass delta change (c).

No between or within group differences were observed for fat mass (*p* > 0.05, placebo: mean_diff_ 0.3 kg, +0.3%; krill oil: mean_diff_ −0.6 kg, −3.6%, Figure 4).

#### 3.2.2. Muscle Strength

**Figure 5:**
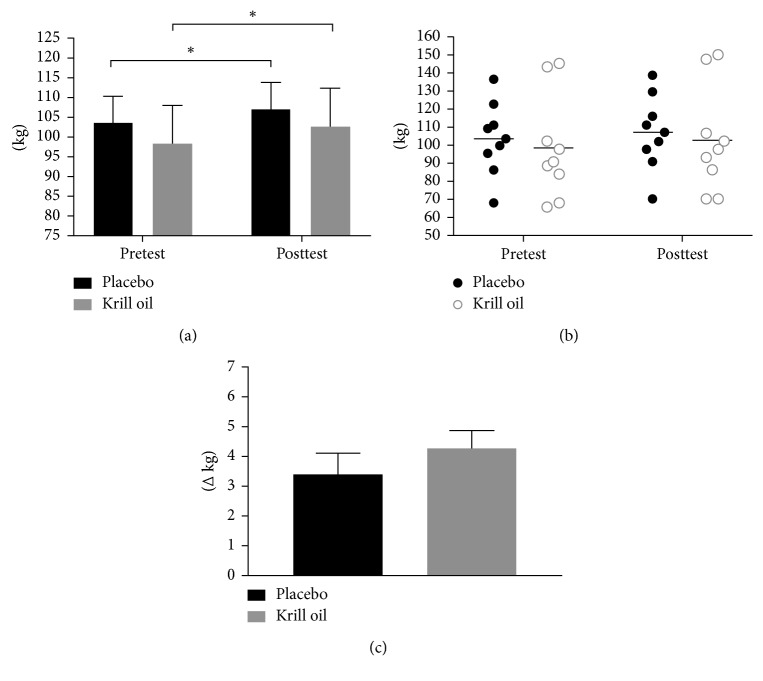
Summary of group bench-press performance (a), individual bench-press performance (b), and bench-press performance delta change (c) (^∗^significant time effect whereby each group increased from baseline (*p* < 0.001)).

**Figure 6:**
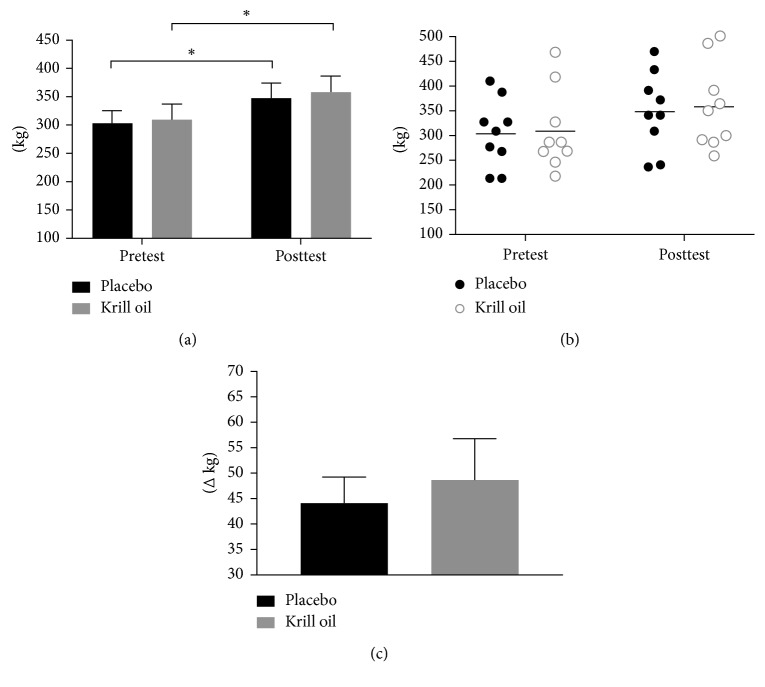
Summary of group leg-press performance (a) and individual leg-press performance (b), and leg-press performance delta change (c) (^∗^significant time effect whereby each group increased from baseline (*p* < 0.001)).

A significant main time effect was demonstrated whereby both conditions increased performance on bench press (*p* < 0.0001, placebo: mean_diff_ 3.4 kg, +3.5%; group krill oil: mean_diff_ 4.2 kg, +4.3%, Figure 5) and leg press (*p* < 0.0001, placebo: mean_diff_ 44.3 kg, +15.8%; krill oil: mean_diff_ 49.0 kg, +15.8%, Figure 6) from pre-to posttesting.

#### 3.2.3. Perceptual Measures

**Figure 7:**
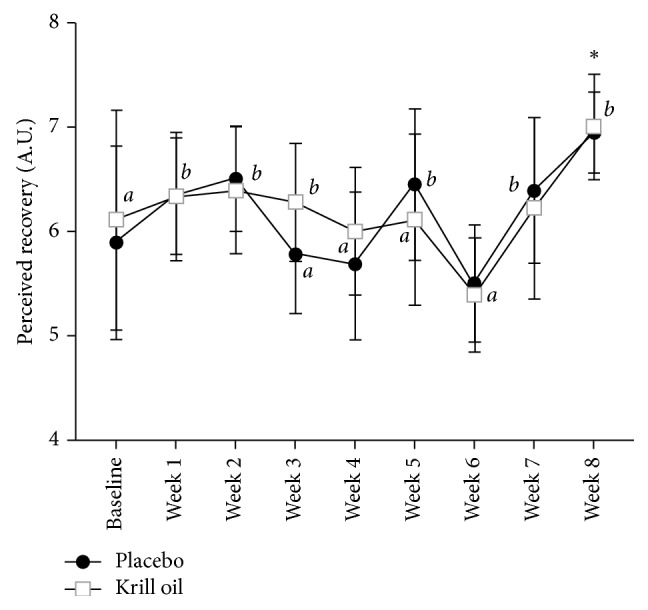
Summary plot for perceived recovery scale weekly average (^∗^significantly higher than baseline, *a* = significantly lower than week 8 (*p* < 0.05), and *b* = significantly higher than week 6 (*p* < 0.05)).

A main time effect was demonstrated for perceived recovery (*p* < 0.001). Placebo had a significantly higher perceived recovery at week 8 compared to baseline and weeks 3, 4, and 6; while weeks 2, 5, and 7 were also higher than week 6. Krill oil had a significantly higher perceived recovery at week 8 compared to baseline and weeks 4, 5, and 6; while weeks 1, 2, and 3 were also higher than week 6 (Figure 7). There were no between group differences for perceived recovery at any time point (Figure 7).

#### 3.2.4. Stroop Test

A main time effect was demonstrated for the Stroop test completion time (*p*=0.007), and post hoc analysis indicated that each group demonstrated strong trends for within-group differences from pre- to posttesting (placebo, *p*=0.087; krill oil, *p*=0.085) (Table 3).

#### 3.2.5. Blood Safety Markers

There were no within or between group differences for markers on the comprehensive metabolic panel (Table 4), complete blood count (Table 5), or urine analysis (not depicted) (*p* > 0.05).

## 4. Discussion

### 4.1. mTOR Activation

Previous studies have shown that soy-derived phosphatidic acid (PA), lyso-PA, or phosphatidylserine (PS) can stimulate a robust increase in mTOR signaling; however, soy-derived PC was not effective [21]. While egg-derived PA increases mTOR signaling, its effects are inferior to soy-derived PA. Egg-derived PA has a different fatty acid composition in comparison to soy-derived PA, containing omega-3 fatty acids, such as DHA or EPA, indicating that the fatty acid composition of the phospholipid influences mTOR signaling and omega-3s likely attenuates the stimulatory effect. Krill oil is a complex mixture of different ingredients with PC being the main phospholipid. Krill oil-PC, like egg-derived phospholipids, is rich in omega-3 fatty acids which suggest that other ingredients in krill oil such as PA, PS, or even astaxanthin or the combination of these ingredients, might be responsible for the effect on mTOR signaling, as astaxanthin has recently been shown to increase mTOR expressions in mice [25].

### 4.2. Body Composition and Athletic Performance

Phospholipids, including PC, PS, or PA, have previously been shown to improve athletic performance [21, 26]. PS has been shown to reduce exercise-induced increases in the catabolic hormone cortisol [27] and reduce muscle soreness [28]. PA enhances exercise-stimulated increases in lean body mass through activation of the mTOR pathway [21], and PC has been shown to maintain choline concentration during exercise [29]. Muscle contractions are induced by signals carried along cholinergic nerves to the muscle fiber, and strenuous exercise has been reported to result in decreased choline concentrations, which negatively influence the rates in which the neurotransmitter acetylcholine is synthesized and released. Maintaining choline levels during exercise has been linked to an increase in athletic performance. 3 g of krill oil provides 1.3 g of phospholipids. The phospholipids in krill oil consist of 90.5% choline containing phospholipids (76.1% PC, 7.0% alkyl acyl PC, 6.5% lyso-PC, and 0.9% lyso-alkyl acyl PC), 8.3% ethanolamine containing phospholipids (3.2% phosphatidylethanolamine (PE), 2.3% cardiolipin n-acetyl PE (CL/NAPE), 1.9% alkyl acyl PE, 0.7% lyso-PE, and 0.2% lyso-alkyl acyl PE), 0.7% phosphatidylinositol (PI), and 0.6% PS [30]. The 1.2 g phosphatidylcholine from 3 g of krill oil provides 160 mg choline, and krill oil contains 7.8 mg PS and 0.54 mg of astaxanthin. The concentration of all individual active ingredients in 3 g krill oil is lower than the currently established individual effective dose (600 mg of PS to lower cortisol levels, 4 mg of astaxanthin, or 0.2 g per kg body weight of PC). A potential synergy of the different active ingredients at low doses could not be established in this trial.

While lean body mass significantly increased in the krill oil group (+1.4 kg, +2.1%) we observed no statistically significant difference in comparison to the control group (+0.3 kg, +0.5%). The increase in lean body mass was matched by an increase in muscle strength in the krill oil group (bench press +4.3 kg (+4.4%); leg press: +48.9 kg (+15.8%)); however, the increase was no different from the control group (+3.4 kg (+3.3%); leg press: +44.2 kg (+14.6%)). Perceived recovery significantly increased in the krill oil group (+15%) at the end of the trial in comparison to baseline; however, the results were not statistically different from the control group (+14%). Perceived recovery was the lowest in both groups in week 6, and subjects seem to adapt to recovering to the training stimulus by the end of the study. While the training protocol was challenging enough to see significant increases in strength, the training stimulus or the recovery periods might not have been challenging enough to elicit differences in perceived recovery. Our results contrast with previous studies showing significant improvements of postexercise muscle soreness with omega-3 supplementation [4–8], which might be due to the lower dose of omega-3s administered in our study, 963 mg for 8 weeks versus 2.4–6 g/d for 1 to 8 weeks, or the difference in training stimulus and recovery periods. Based on a post hoc power analysis the sample size needed to reach statistical significance would be between 34 and 84 subjects for changes in lean body mass, depending on the level of statistical power (i.e., 50% to 90% probability of detecting a significant difference at an alpha level of 0.05). Future studies should investigate the activation of mTOR *in vivo* through a muscular biopsy.

### 4.3. Cognition

While long-chain omega-3 polyunsaturated fatty acid supplementation has been linked to improved cardiovascular health [31], the body of published clinical trials on the potential cognitive benefits that may result from improving long-chain omega-3 fatty acid intakes in healthy young adults is limited. Omega-3 polyunsaturated fatty acids may improve brain functions by facilitating vasodilatation and perfusion [32]. DHA supplementation in subjects with very low dietary intakes of DHA and EPA showed significant improvement in working memory, completing a working memory task 20% faster relative to placebo [33]. A study investigating which long-chain omega-3 polyunsaturated fatty acid, EPA-rich or DHA-rich, might improve cognitive performance and functional brain activation in young adults showed that EPA-rich omega-3 fatty acids showed greater cognitive performance in a Stroop color-word test [34]. While krill oil is a EPA-rich source of omega-3 fatty acids, the completion time in the Stroop color-word test improved after the supplementation period in the krill oil group as well as in the control subjects. This may be due to a familiarization effect. The participants knew the situation and tasks during the second trial and, therefore, may have attained better results.

## 5. Conclusion

Krill oil activates mTOR signaling. Krill oil supplementation in athletes is safe, and while no significant effects on cognition and strength were observed, its effects on body composition in combination with resistance exercise deserves further research.

## Figures and Tables

**Table 1:** Subject characteristic data.

	Age (yrs)	Training age (yrs)	Body mass (kg)	Height (cm)
Placebo (*n*=9)	22.0 ± 0.6	3.0 ± 0.2	79.9 ± 3.9	179.3 ± 3.1
Krill oil (*n*=9)	22.3 ± 0.4	3.1 ± 0.7	82.9 ± 5.0	178.8 ± 2.3

**Table 2:** Training Protocol.

Weekly Schedule					

Weight Training	Week	1^E^	2^H^	3^E^	4^H^
	Hypertrophy/endurance:	3 sets/12 repetitions 60s rest	3 sets/8 repetitions 90 s rest	3 sets/15 repetitions 60s rest	3 sets/10 repetitions 90s rest
	Strength:	3–5 sets/5 repetitions 3–5 min rest	3–5 sets/4 repetitions 3–5 min rest	3–5 sets/3 repetitions 3–5 min rest	3–5 sets/5 repetitions 3–5 min rest
	**Week**	**5** ^**E**^	**6** ^**H**^	**7** ^**E**^	**8** ^**H**^
	Hypertrophy/endurance:	3 sets/12 repetitions 90 s rest	3 sets/8 repetitions 90s rest	3 sets/15 repetitions 60s rest	3 sets/10 repetitions 90s rest
	Strength:	3–5 sets/ 4 repetitions 3–5 min rest	3–5 sets/4 repetitions 3–5 min rest	3–5 sets/ 3 repetitions 3–5 min rest	3–5 sets/ 3 repetitions 3–5 min rest

Exercise Selection					

Monday	Tuesday	Wednesday	Thursday	Friday	Saturday

*Strength*	*Rest*	*Hypertrophy/endurance*	*Hypertrophy/endurance*	*Rest*	*Strength*
Leg press		Bench press	Squat		Bench press
Squat		BB row	Deadlift		BB row
Glute-ham raise		BB shoulder press	Leg press		BB shoulder press
		Pull-ups	Glute-ham raise		Pull-ups
		DB press	Seated leg curl		
		Incline DB press	Lying leg curl		
		Side lateral raise	Leg ext.		
		Preacher curl	Calf raise		
		Overhead tricep ext.			
		Cable fly			
		Hammer curl			
		Tricep pushdown			
		Cable press			

**Table 3:** Stroop test results.

	Placebo		Krill oil	
	Pre	Post	Pre	Post
Congruent	5.0 ± 0.0	5.0 ± 0.0	5.0 ± 0.0	5.0 ± 0.0
Incongruent	14.7 ± 0.2	14.8 ± 0.2	14.8 ± 0.2	14.8 ± 0.2
Completion time	32.6 ± 6.7	17.2 ± 2.3 ^	33.8 ± 6.1	18.3 ± 2.1 ^

^Trend for within-group significant differences from pretest (*p* < 0.1).

**Table 4:** Comprehensive metabolic panel.

	Placebo		Krill oil		*G* × *Tp* value
	Pre	Post	Pre	Post	
Glucose (mg/dL)	84 ± 2	86 ± 4	91 ± 3	90 ± 4	0.689
BUN (mg/dL)	13 ± 1	13 ± 1	13 ± 2	15 ± 2	0.200
Creatinine (mg/dL)	1.07 ± 0.1	1.05 ± 0.1	1.06 ± 0.1	1.00 ± 0.1	0.523
Total protein (g/dL)	7.1 ± 0.1	7.1 ± 0.1	7.2 ± 0.1	7.0 ± 0.1	0.114
Albumin (g/dL)	4.6 ± 0.1	4.5 ± 0.1	4.8 ± 0.1	4.6 ± 0.1	0.227
Total bilirubin (mg/dL)	0.4 ± 0.1	0.3 ± 0.1	0.6 ± 0.3	0.5 ± 0.3	0.510
Sodium (mmol/L)	141 ± 1	141 ± 1	142 ± 1	141 ± 1	0.339
Potassium (mmol/L)	4.3 ± 0.1	4.2 ± 0.1	4.3 ± 0.1	4.3 ± 0.1	0.328
Chloride (mmol/L)	100 ± 1	101 ± 1	101 ± 1	101 ± 1	0.939
CO_2_ (mmol/L)	23 ± 1	22 ± 1	22 ± 1	22 ± 1	0.791
ALP (IU/L)	73 ± 6	75 ± 6	75 ± 5	70 ± 6	0.075
AST (IU/L)	24 ± 5	22 ± 2	20 ± 1	17 ± 1	0.827
ALT (IU/L)	22 ± 5	19 ± 3	19 ± 1	17 ± 2	0.754

Values are reported as mean ± standard deviation; BUN, blood urea nitrogen; CO_2_, carbon dioxide; ALP, alkaline phosphatase; AST, aspartate aminotransferase; ALT, alanine transaminase.

**Table 5:** Complete blood count panel

	Placebo		Krill oil		*G* × *Tp* value
	Pre	Post	Pre	Post	
WBC (K/*µ*L)	5.6 ± 0.2	5.5 ± 0.2	5.7 ± 0.2	5.5 ± 0.3	0.586
RBC (M/*µ*L)	5.01 ± 0.14	5.12 ± 0.12	5.26 ± 0.08	5.08 ± 0.09	0.114
Hemoglobin (g/dL)	15.1 ± 0.4	15.4 ± 0.2	14.7 ± 1.1	15.3 ± 0.3	0.785
Hematocrit (%)	44.6 ± 1.2	45.3 ± 0.7	46.9 ± 0.9	45.7 ± 1.1	0.160
MVC (fL)	89.1 ± 1.6	89.2 ± 1.1	88.8 ± 1.3	90.4 ± 1.2	0.414
MCH (pg)	30.2 ± 0.7	29.9 ± 0.5	30.0 ± 0.6	30.0 ± 0.4	0.999
MCHC (g/dL)	33.4 ± 0.2	33.2 ± 0.3	33.8 ± 0.3	33.3 ± 0.2	0.481
Platelets (K/*µ*L)	228 ± 10	221 ± 10	232 ± 11	219 ± 10	0.431
Neutrophils (K/*µ*L)	2.8 ± 0.2	2.9 ± 0.2	3.1 ± 0.2	2.8 ± 0.2	0.197
Lymphocytes (K/*µ*L)	2.1 ± 0.1	2.0 ± 0.1	2.0 ± 0.1	2.0 ± 0.1	0.999
Monocytes (K/*µ*L)	0.5 ± 0.1	0.5 ± 0.1	0.5 ± 0.1	0.5 ± 0.2	0.739
Eosinophils (K/*µ*L)	0.1 ± 0.1	0.2 ± 0.1	0.1 ± 0.1	0.1 ± 0.1	0.461
Basophils (K/*µ*L)	0.0 ± 0.0	0.0 ± 0.0	0.0 ± 0.0	0.0 ± 0.0	0.555

Values are reported as mean ± standard deviation; WBC, white blood cells; RBC,  red blood cells; MVC, mean cell volume; MCH, mean cell hemoglobin; MCHC,  mean cell hemoglobin concentration.
